# Association between dietary inflammation index and female infertility from National Health and Nutrition Examination Survey: 2013-2018

**DOI:** 10.3389/fendo.2024.1309492

**Published:** 2024-05-02

**Authors:** Jie Qi, Yujie Su, Huanhuan Zhang, Yanan Ren

**Affiliations:** ^1^ Department of Gynecology, Hebei General Hospital, Shijiazhuang, China; ^2^ Department of Gynecology, Shandong Provincial Maternal and Child Health Care Hospital Affiliated to Qingdao University, Jinan, Shandong, China; ^3^ Department of Anesthesiology, Hebei General Hospital, Shijiazhuang, Hebei, China

**Keywords:** infertility, dietary inflammatory index, nutrition, National Health and Nutrition Examination Survey, RCS

## Abstract

**Objective:**

To investigate the relationship between dietary inflammatory index (DII) scores and infertility in US adults aged 18 to 45.

**Methods:**

Data were gathered from the 2013-2018 National Health and Nutrition Examination Survey (NHANES). In total, 3496 women were included in the study. To examine the relationship between DII, EDII and infertility, a weighted multivariable logistic regression analysis using continuous factors or categorical variables grouped by quartiles was conducted. Using subgroup analysis stratified based on DII and infertility features, the association between DII and infertility has been further studied. In order to determine whether there was a nonlinear relationship between DII and infertility, restricted cubic spline (RCS) analysis was carried out.

**Results:**

For statistical analysis, a total of 3496 individuals — 367 patients with infertility and 3129 persons without infertility — were included. A multivariable logistic regression study revealed a positive relationship between DII and infertility. A significant difference in subgroup analysis was shown in age group and race, although RCS analysis demonstrated nonlinear relationship between the DII and infertility.

**Conclusion:**

For participants aged 18-45 years, higher DII scores were positively correlated with infertility. In addition, anti-inflammatory diets might improve infertility outcomes.

## Introduction

Infertility is the inability to conceive and reproduce due to a variety of etiologic factors. Infertility is defined as the failure to conceive after at least 12 months of uncontraceptive sexual intercourse (1–4). It is a serious global public health problem that is estimated to affect approximately 15% of the world’s population, with nearly 48.5 million (45 million, 52.6 million) couples experiencing infertility globally (5). Infertility is an important component of reproductive health. The inability to have children has a significant impact on the physical and mental health of those women, leading to distress and depression (6, 7), which is also associated with population decline and low fertility rates (8). The factors that lead to infertility are complex — several common diseases may affect female infertility, such as premature ovarian insufficiency (1, 9), polycystic ovary syndrome (10), endometriosis (11, 12), uterine fibroids (13), and endometrial polyps (9). However, in addition to these common diseases, a number of factors related to lifestyle have gained prominence in recent years, such as diet and chronic inflammation, and the diets of modern people also bring about an inflammatory response of the body (14).

Inflammation plays an important role in reproduction. Studies have reported that patients with polycystic ovary syndrome have higher levels of C-reactive protein (CRP), interleukin 18 (IL-18), interleukin-1β (IL-1β), tumor necrosis factor-α (TNF-α), interleukin 6 (IL-6), white blood cell counts (WBCs), monocyte chemoattractant protein-1 (MCP-1), and macrophage inflammatory protein- 1α (MIP-1α) (15–17). In patients with endometriosis, there was a trend of increased inflammatory indices, which confirmed the immunologic alterations in these diseases (18, 19). There was an imbalance between anti-inflammatory and pro-inflammatory cytokines in patients with ovarian failure, so inflammation was closely associated with premature ovarian insufficiency (20, 21). The Dietary Inflammatory Index (DII) is a scoring system that evaluates the inflammatory potential of the diet, with higher scores being more favorable to inflammation (22). The DII was initially developed by Shivappa et al. (22), and it encompasses 45 food parameters including various micro- and macronutrients, spices, and flavonoids. Each of these parameters is assigned a score based on its pro- or anti-inflammatory properties as validated by extensive research studies. However, DII does not account for total energy intake, which can be a confounding factor, as total energy intake may influence the overall inflammatory potential of the diet. To overcome this limitation, Shivappa et al. (23), later proposed the E-DII, which adjusts DII scores for total energy intake using the residual method.Both DII and E-DII have been utilized as tools in numerous epidemiological studies to investigate the relationship between diet-induced inflammation and various health outcomes, including cancer, cardiovascular diseases, metabolic syndrome, and mortality, among others. Previous studies have shown significant associations between DII and risks of obesity and neoplasia (24), but to our knowledge, very little research has reported a correlation between DII and infertility. This represents a significant gap in our knowledge, as understanding the potential association between dietary inflammation and infertility could have important implications for disease prevention and treatment strategies. In our study, we used cross-sectional analyses to investigate women with infertility from 2013-2018 in an attempt to get to the bottom of their relationships.

## Materials and methods

**Figure 1:**
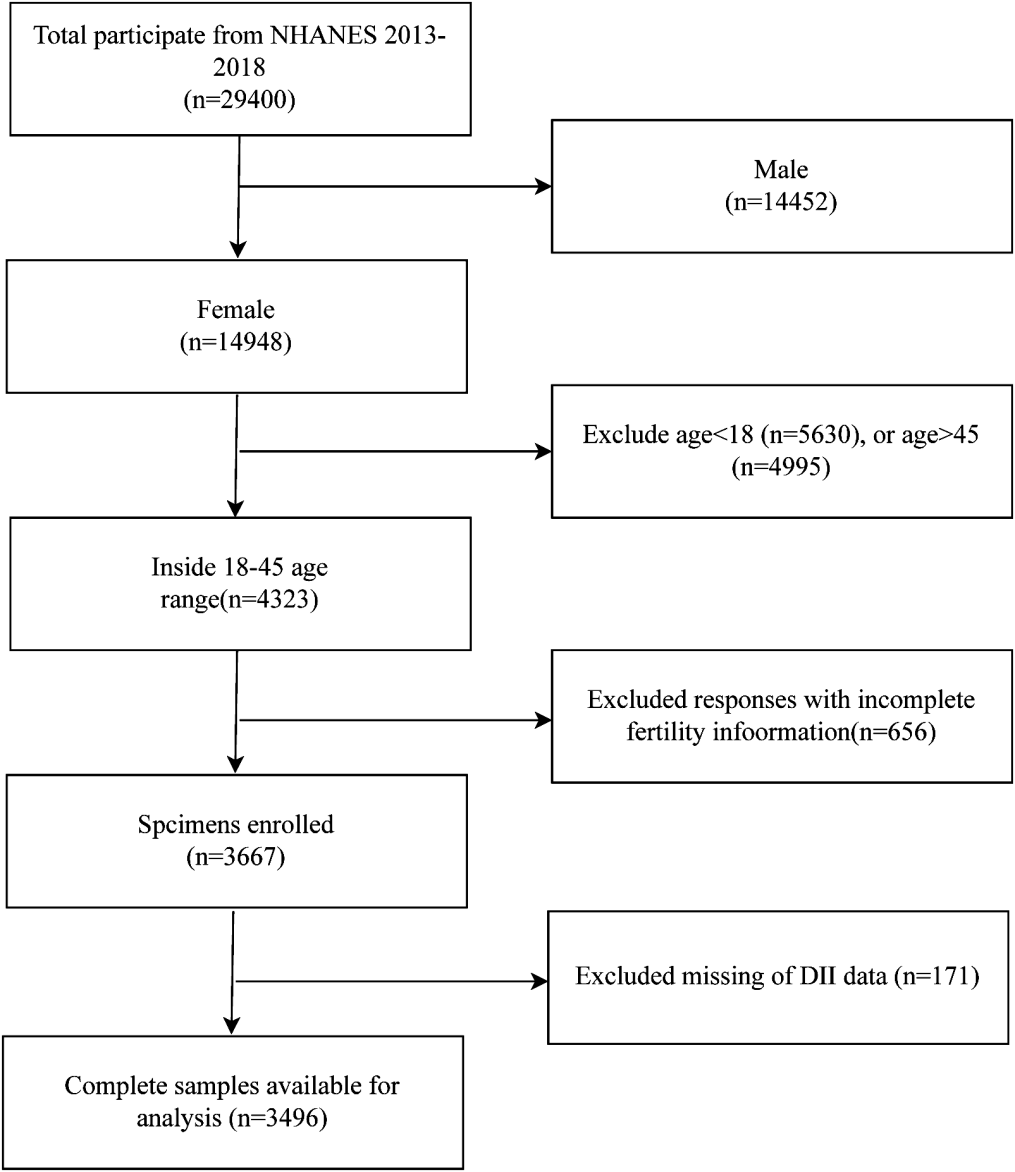
The flow chart of sample selection from NHANES 2013-2018.


The NHANES database is a public program that assesses the health and nutrition of Americans. It is presented through questionnaires, laboratory data, and physical measurements. We selected data from this database for the years 2013-2018. In total, there are 29,400 subjects. We excluded men (14,452), those under 18 years of age (5,630), those over 45 years of age (4,995), women with no information on infertility (656), and those with no DII data (171) were excluded, resulting in 3,496 subjects being included in our study (**Figure 1**). The formula of sample size calculation:

$$\begin{array}{c} \mathrm{Final}\;\mathrm{probability}=(\mathrm{Pr}\left(\mathrm{PSU}\;\mathrm{is}\;\mathrm{selected}\right)\\ \times \mathrm{Pr}\left(\mathrm{segment}\;\mathrm{of}\;\mathrm{the}\;\mathrm{PSU}\;\mathrm{is}\;\mathrm{selected}\right)\\ \times \mathrm{Pr}\left(\mathrm{household}\;\mathrm{is}\;\mathrm{selected}\right)\\ \times \mathrm{Pr}\left(\mathrm{individual}\;\mathrm{is}\;\mathrm{selected}\right)) \end{array}$$

### Measurement of DII and EDII

Individual dietary data were obtained through by the average of the first in-person collection in the Mobile Examination Center (MEC) and a telephone interview 3-8 days later (second 24-hour dietary recall interview). In NHANES, 27 foods were available for DII calculation: 1: carbohydrates; 2: protein; 3: total fat; 4: alcohol; 5: fiber; 6: cholesterol; 7: saturated fat; 8: MUFA; 9: PUFA; 10: n-3 fatty acids eicosapentaenoic (20:5), docosapentaenoic (22:5), docosahexaenoic (22:6); 11: n-6 fatty acids, octadecadienoic (18:2), octadecatrienoic (18:3), octadecatetraenoic (18:4), eicosatetraenoic (20:4); 12: niacin; 13: vitaminA; 14: thiamin (vitamin B1); 15: riboflavin(vitamin B2); 16: vitamin B6; 17: vitamin B12; 18: vitamin C; 19: vitamin E; 20: Fe; 21: Mg; 22: zinc; 23: selenium; 24: folic acid; 25: beta-carotene; 26:caffeine; 27: energy. The calculation of the DII has been reported in the literature (22), and this dietary database has means and standard deviations (SD) for a total of 45 food parameters.DII was calculated by subtracting the mean of their total number from the raw data in the database and dividing by the standard deviation of the parameter to obtain z. z was converted to a percentile score by doubling and subtracting 1 (from -1 to +1, centered on 0). The result was multiplied by the corresponding literature-derived inflammatory effect score for each food parameter. Finally, the overall DII score for each individual is the sum of the DII scores for each specific food parameter. Higher DII scores indicate a more pro-inflammatory diet (25). The procedure for calculating the E-DII was the same as for calculating the DII, which was designed to control the effects of total energy intake, with energy-adjusted DII scores calculated for each 1,000 calories of food consumed (23, 26) (using the energy-standardized version of the World Data Bank). This adjustment is usually done using the residual method, where the residuals from a regression of DII on total energy intake are used to obtain an energy-independent DII score.

### Main outcomes

The outcome variable infertility was among the reproductive health questionnaires, RHQ074. The question for this variable was “Have you ever tried to get pregnant for at least one year without getting pregnant?” If the answer was “Yes”, you were considered infertile.

### Other variables

Demographic variables included age, race (Mexican American, Non-Hispanic Black, Non-Hispanic White, Other Hispanic, Other Race), marital status (non-single, single), education level (high school or below, high school, high school level or higher).

#### Comorbidities

Diagnostic criteria for diabetes were based on:(1) physician diagnosis of diabetes, (2) glycated hemoglobin HbA1c (%) >6.5, (3) fasting blood sugar (mmol/L) >7.0.4, (4) random blood sugar (mmol/L) ≥11.1 and (5) 2-hour OGTT blood sugar (mmol/L) ≥11.1,(6) Use of diabetes medications or insulin. Hypertension diagnosis was based on: (1) doctors’ diagnosis of high blood pressure, (2) use of antihypertensive medications, and (3) abnormal blood pressure readings (>=3 times).

#### Some other variables

Diagnostic criteria for alcohol use were as follows: (1) Never: <12 drinks in a lifetime. (2) Former: ≥12 drinks in 1 year and no drinking last year, or no drinking last year but ≥12 drinks in a lifetime. (3) Yes: those except the above two. There were three categories of smoking: never, former, and now: (1) Never: smoked <100 cigarettes in a lifetime; (2) former: smoked >100 cigarettes in a lifetime and not currently smoking; (3) Now: smoked >100 cigarettes in a lifetime and smoking some days or every day. There was also physical activity, insurance status, and pregnancy status.

### Statistical analysis

For the statistical analysis of this study, NHANES took survey weights into account. Continuous variables are presented as mean ± SD, and categorical variables are presented as percentages. Specifically, multivariate logistic regression was used to assess the association between DII, E-DII and infertility while adjusting for covariates. To explore the relationship between DII, E-DII and infertility, DII and E-DII were divided by continuous variables into 4 subgroups respectively — categorical variables were used to calculate the differences between different DII and E-DII. In Model 1, adjustments were made for age, marital status and BMI. In Model 2, adjustments were made for age, household income ratio, BMI, sedentary time, race, divorce status, education, smoking, alcohol use, diabetes, hypertension, previous pregnancy, outdoor exercise intensity, and insurance. To further explore the relationship between DII and infertility, subgroup analyses were conducted. Additionally, we utilized a restricted cubic spline (RCS) to account for potential non-linear relationships between DII and infertility, which places knots at the 5th, 35th, 65th, and 95th percentiles of the predictor distribution. RCS provides flexibility by allowing the function to change at specific values of the predictor, known as knots. The statistical software packages R (http://www.R-project.org) and Empower Stats (http://www.empowerstats.com) were used for analysis. P < 0.05 was considered statistically significant.

## Results

### Demographic and clinical characteristics

The study comprised 3496 participants with an infertile cohort (n=367) showing a significantly higher mean age (35.45 vs. 30.91 years, p<0.0001) and BMI. Presence of comorbidities (diabetes and hypertension) and lifestyle habits (drinking and smoking) were more prevalent in the infertile group (p<0.05). The analysis also involved diverse ethnic backgrounds i.e., Mexican Americans, other Hispanic, non-Hispanic white, non-Hispanic black, and Non-Hispanic Asian (**Table 1**).

**Table 1:** Baseline characteristics of participant.

Characteristic	infertility	
	negative	positive
Age, mean(sd),years	30.905 (30.484,31.327)	35.451 (34.550,36.352)
BMI,mean(sd),kg/m2	28.964 (28.487,29.440)	32.288 (31.001,33.575)
Poverty-to-income ratio, mean(sd)	2.633 (2.512,2.755)	2.832 (2.612,3.052)
Sedentary Time,mean (sd) (min)	383.282 (373.250,393.314)	400.270 (375.493,425.047)
DII	1.752 (1.626,1.877)	2.100 (1.891,2.310)
EDII	1.414 (1.298,1.530)	1.730 (1.438,2.022)
Race/Ethnicity n(%)		
Mexican American	12.059 (9.538,15.136)	10.132 (6.626,15.191)
Other Hispanic	13.679 (11.053,16.811)	12.112 (9.178,15.820)
Non-Hispanic White	55.789 (50.938,60.533)	63.170 (55.382,70.327)
Non-Hispanic Black	8.052 (6.577,9.824)	5.811 (3.563,9.339)
Non-Hispanic Asian	10.420 (8.912,12.149)	8.776 (6.346,12.015)
Education level n(%)		
Less than high school	3.134 (2.331,4.201)	2.190 (1.136,4.178)
High school	30.451 (27.513,33.557)	27.962 (22.940,33.605)
More than high school	66.416 (62.929,69.732)	69.848 (63.963,75.146)
Marital status n(%)		
Married and living with partner	57.423 (54.671,60.131)	78.186 (72.788,82.766)
Living alone	42.577 (39.869,45.329)	21.814 (17.234,27.212)
Smoking n(%)		
never	70.506 (68.086,72.816)	61.724 (55.602,67.496)
former	11.634 (10.149,13.305)	14.324 (10.318,19.545)
now	17.860 (16.101,19.766)	23.952 (18.162,30.891)
drinking n(%)		
never	15.705 (13.438,18.273)	10.954 (6.826,17.119)
former	4.686 (3.856,5.685)	8.728 (5.646,13.256)
now	79.609 (76.406,82.476)	80.318 (73.579,85.673)
Diabetes n(%)		
Yes	5.616 (4.784,6.584)	11.267 (8.679,14.505)
No	94.384 (93.416,95.216)	88.733 (85.495,91.321)
Hypertension,n(%)		
yes	13.569 (12.094,15.192)	23.763 (18.808,29.548)
no	86.431 (84.808,87.906)	76.237 (70.452,81.192)
Vigorous recreational activities, n (%)		
yes	17.748 (15.761,19.927)	17.125 (12.668,22.742)
no	82.252 (80.073,84.239)	82.875 (77.258,87.332)
Moderate recreational activities, n (%)		
yes	43.369 (41.006,45.763)	41.970 (35.254,48.998)
no	56.631 (54.237,58.994)	58.030 (51.002,64.746)
Health insurance, n (%)		
none	17.933 (16.225,19.778)	21.240 (16.084,27.507)
prirate	58.111 (55.121,61.041)	59.032 (52.626,65.145)
public	23.957 (21.439,26.669)	19.729 (15.637,24.579)
Ever been pregant		
yes	66.841 (63.710,69.830)	86.153 (81.916,89.525)
no	33.159 (30.170,36.290)	13.847 (10.475,18.084)

For continuous variables: P-value was by survey-weighted linear regression. For categorical variables: P-value was by survey-weighted Chi-square test. BMI, body mass index; NHANES, National Health, and Nutrition Examination Survey; SD, standard deviation.

#### Dietary inflammatory indexand infertility

A significantly higher DII was observed in the infertile group compared to controls (2.10 vs 1.75). Furthermore, each unit increase in DII was associated with a 10% increased odds of infertility. When DII was categorized into quartiles, the highest quartile (Q4) was associated with a 59% higher risk of infertility compared to the lowest quartile (Q1) (**Table 2**).

**Table 2:** The association between infertility and DII 、E-DII.

Exposure	Non-adjusted model OR,95%CI	Minimally-adjusted model OR,95%CI	Fully-adjusted model OR,95%CI
DII	1.11(1.03,1.12)0.007	1.12(1.04, 1.20)0.007	1.10(1.01,1.19)0.034
DII			
Q1	Ref	Ref	Ref
Q2	1.02(0.66,1.56)0.945	1.05(0.65,1.69)0.840	1.05(0.65,1.70)0.829
Q3	1.20 (0.84,1.72)0.313	1.23(0.868,1.75)0.250	1.02(0.70,1.49)0.900
Q4	1.54(1.053, 2.24)0.031	1.71(1.14 ,2.58)0.014	1.59(1.03,2.45)0.045
P for trend	0.023	0.011	0.068
Exposure	Non-adjusted model OR,95%CI	Minimally-adjusted model OR,95%CI	Fully-adjusted model OR,95%CI
E-DII	1.05(1.00,1.09)0.034	1.06 (1.01,1.11)0.019	1.04 (0.99,1.08)0.109
E-DII			
Q1	Ref	Ref	Ref
Q2	1.06(0.65,1.72)0.823	1.14 (0.69,1.88)0.620	1.07 (0.630, 1.82)0.804
Q3	1.35(0.88,2.07)0.184	1.47(0.95,2.29)0.093	1.40 (0.86, 2.27)0.188
Q4	1.42(0.99,2.04)0.066	1.46 (0.99,2.16)0.060	1.31 (0.89, 1.92)0.177
P for trend	0.025	0.021	0.086

Non-adjusted model: no covariates were adjusted for.

Minimally-adjusted model: we only adjusted for age 、Marital status and BMI.

Fully-adjusted model: we adjusted for all covariates presented in **Table 1**.

### Empirical dietary inflammatory index and infertility

EDII was significantly higher in the infertile group compared to the control group (1.73 vs 1.41). An increasing trend in the risk of infertility was observed with increasing levels of EDII (**Table 2**).

### Stratified analysis

**Figure 2:**
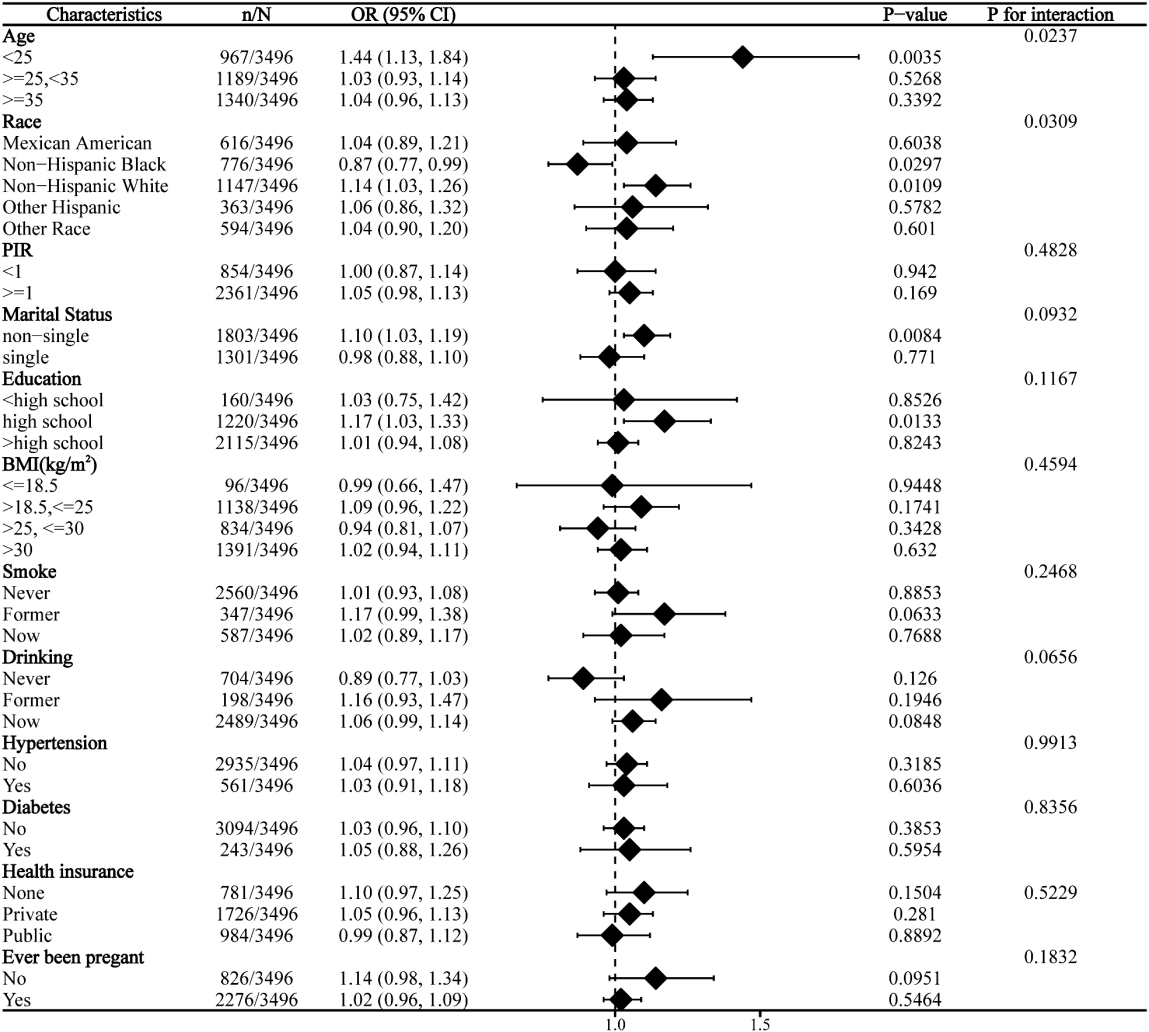
Subgroup analysis for the association between DII and infertility.

Subgroup analysis revealed significant interaction effects of age and race on the relationship between DII and infertility. No significant interaction was observed across other strata (**Figure 2**).

#### Nonlinear relationship

**Figure 3:**
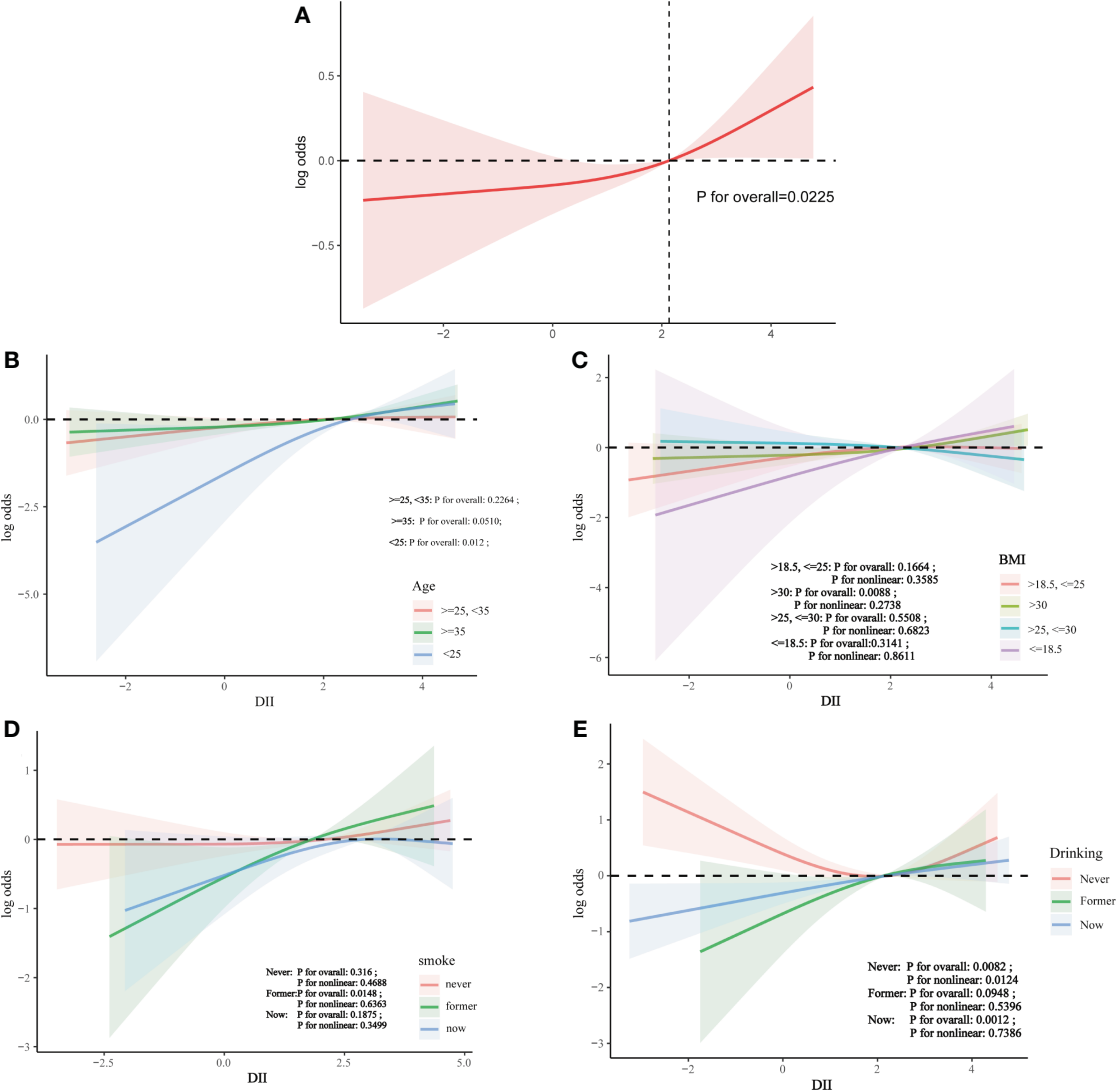
The association between DII and infertility. **(A)** Solid line plot of curve fitting with DII and infertility as variables.The red line indicates the smooth curve fit between the variables. The 95% confidence interval of the fit is shown by the red bar. **(B)** The association between DII and infertilit stratified by age. **(C)** The association between DII and infertilit stratified by BMI. **(D)** The association between DII and infertilit stratified by smoke. **(E)** The association between DII and infertilit stratified by drinking.

Analysis using restricted cubic splines showed a linear relationship between DII and infertility risk, with no evidence of nonlinearity (**Figure 3**).

## Discussion

This was a cross-sectional study of 3496 women aged 18-45 years, from which it was observed that there was a positive correlation between DII and infertility, suggesting that consumption of a pro-inflammatory diet increased the risk of infertility. After adjusting for covariates, the positive association between DII and infertility remained. However, after stratification, the positive association was affected by age and race. In the final curvilinear relationship, there was no nonlinear association between DII and infertility.

To our knowledge, there are few studies to explore the relationship between DII and infertility. A RaNCD cohort study verified the association between infertility and the quality of diet in women, the results showed the odds ratio of infertility in the pro-inflammatory diet was 1.76 times higher than in the anti-inflammatory diet of DII (95% CI: 1.57-2.02) (27). Its results are consistent with ours. There have been previous studies on the relationship between diet and infertility. One study has confirmed that a Mediterranean nutritional pattern reduces the risk of weight gain and insulin resistance, which may be responsible for increased pregnancy (28, 29). In 2007, a prospective cohort study created a “fertility diet” pattern that included a lower intake of animal proteins and higher availability of plant proteins. The results suggested that increased adherence to a “fertility diet” could improve infertility caused by ovulation disorders (30, 31). It is well known that inflammation occurs throughout almost the entire reproductive process, from ovulation, implantation, and fertilization of the egg to pregnancy. Inflammation is a normal process of injury and infection, but prolonged inflammation can impair fertility. Inflammation can damage the endometrium (1, 32), trigger oxidative stress that impairs folliculogenesis (33), and alter blood coagulation leading to thrombosis (13). A prospective study showed that chronic endometritis affected homeostatic imbalance in patients with endometrial fibrosis and was associated with a higher incidence of adhesions, thus leading to reproductive failure.

The odds of infertility are increased with higher DII scores, and the exact mechanism of this positive association remains unclear. However, high DII has a modulating effect on the inflammatory process, which can lead to an increase in inflammatory markers including CRP (34, 35), TNF-α (Kwak-Kim, Yang and Gilman-Sachs, 2009) (36), IL-6 (37), and other markers of inflammation, thus adversely affecting reproduction. TNF-α mediates immune and inflammatory responses; in addition, elevated concentrations of TNF-α in peritoneal fluid can directly reduce sperm viability, thereby affecting the entire fertilization and implantation process and exhibiting embryotoxicity (38). In one study in transgenic mice, the number of implantation sites or larval size was reduced in the absence of cytokines, such as CSF-1, GM-CSF, IL-1 and IL-6 (39). There was also a basic study from Michigan, USA, in which mouse oocytes were exposed to IL-6 (50, 100, and 200 ng/mL) for 30 min, as compared with untreated controls. It was found that IL-6 resulted in dose-dependent deterioration of microtubule and chromosome arrangement in the treated oocytes, compared with the untreated group, suggesting that elevated levels of IL-6 might be mediated through a mechanism involving impaired microtubule and chromosome architecture to reduce the fertilizing ability of human oocytes (40). There have also been several studies suggesting that anti-inflammatory diets may improve fertility outcomes. In a prospective study of 18,555 premenopausal women, this anti-inflammatory diet prevented ovulatory infertility by reducing carbohydrate intake and overall dietary glucose load (41). A recent randomized controlled trial investigating a subgroup of 150 overweight adult women with polycystic ovary syndrome found that the anti-inflammatory diet group and the physical activity group had improved menstrual cycles and spontaneous pregnancies, as well as a 7% weight loss, and these effects were not inferior to those observed in the metformin group.

One of the strengths of this study is that it is based on a weighted and representative population with a large base size. It is worth noting that we also performed a curve analysis. However, this study has several limitations. First, it is a cross-sectional analysis, therefore, we cannot determine causality. Second, for the DII the calculations were based on 24 h dietary recalls from the population, which may introduce bias in the data. Finally, for confounders, we merely included those shown in **Table 1**, which is obviously insufficient for the outcome variable. In our paper, we focused on specific infertility risk factors. However, given the complexity of infertility, there are other potential risk factors that may have an impact on the observed indicators, such as environmental factors, genetic factors, as well as endometriosis, and polycystic ovary syndrome.These risk factors may influence the indicators we observe. For example, the basis of infertility caused by polycystic ovary syndrome is chronic inflammation caused by immune metabolism (42).Whilst our study has focused primarily on the DII, we recognise that a comprehensive understanding of the complex mechanisms of infertility requires consideration of a wider range of possible risk factors. Therefore, future research should further explore these additional risk factors and their specific impact on infertility. This will help us to gain a deeper understanding of the causes of infertility and may provide new ideas for treatment.

## Conclusion

This study showed a significant positive correlation between DII scores and infertility, which suggests that there is a positive correlation between a pro-inflammatory diet and the incidence of infertility, and that management with an anti-inflammatory diet decreases the chances of infertility. However, further fundamental research is still needed to explore the potential association between them.

## Data availability statement

The original contributions presented in the study are included in the article/supplementary material, further inquiries can be directed to the corresponding author/s.

## Ethics statement

The studies involving humans were approved by National Center for Health Statistics. The studies were conducted in accordance with the local legislation and institutional requirements. Written informed consent for participation in this study was provided by the participants’ legal guardians/next of kin. Written informed consent was obtained from the individual(s), and minor(s)’ legal guardian/next of kin, for the publication of any potentially identifiable images or data included in this article.

## Author contributions

JQ: Writing – original draft. YS: Writing – review & editing. HZ: Data curation, Writing – review & editing. YR: Funding acquisition, Writing – review & editing.
